# *N*-Acetylcysteine Serves as Substrate of 3-Mercaptopyruvate Sulfurtransferase and Stimulates Sulfide Metabolism in Colon Cancer Cells

**DOI:** 10.3390/cells8080828

**Published:** 2019-08-04

**Authors:** Karim Zuhra, Catarina S. Tomé, Letizia Masi, Giorgio Giardina, Giulia Paulini, Francesca Malagrinò, Elena Forte, João B. Vicente, Alessandro Giuffrè

**Affiliations:** 1 Department of Biochemical Sciences, Sapienza University of Rome, Piazzale Aldo Moro 5, I-00185 Rome, Italy; 2 CNR Institute of Molecular Biology and Pathology, Piazzale Aldo Moro 5, I-00185 Rome, Italy; 3 Instituto de Tecnologia Química e Biológica António Xavier (ITQB NOVA), Avenida da República (EAN), 2780-157 Oeiras, Portugal

**Keywords:** hydrogen sulfide, sulfane sulfur species, enzymatic activity assays, protein expression levels, antioxidants, colorectal cancer

## Abstract

Hydrogen sulfide (H_2_S) is an endogenously produced signaling molecule. The enzymes 3-mercaptopyruvate sulfurtransferase (MST), partly localized in mitochondria, and the inner mitochondrial membrane-associated sulfide:quinone oxidoreductase (SQR), besides being respectively involved in the synthesis and catabolism of H_2_S, generate sulfane sulfur species such as persulfides and polysulfides, currently recognized as mediating some of the H_2_S biological effects. Reprogramming of H_2_S metabolism was reported to support cellular proliferation and energy metabolism in cancer cells. As oxidative stress is a cancer hallmark and *N*-acetylcysteine (NAC) was recently suggested to act as an antioxidant by increasing intracellular levels of sulfane sulfur species, here we evaluated the effect of prolonged exposure to NAC on the H_2_S metabolism of SW480 colon cancer cells. Cells exposed to NAC for 24 h displayed increased expression and activity of MST and SQR. Furthermore, NAC was shown to: (i) persist at detectable levels inside the cells exposed to the drug for up to 24 h and (ii) sustain H_2_S synthesis by human MST more effectively than cysteine, as shown working on the isolated recombinant enzyme. We conclude that prolonged exposure of colon cancer cells to NAC stimulates H_2_S metabolism and that NAC can serve as a substrate for human MST.

## 1. Introduction

Hydrogen sulfide (H_2_S) is an endogenously produced signaling molecule involved in the regulation of several physiological processes, such as blood flow, inflammation, neurotransmission, apoptosis, redox homeostasis, energy metabolism and stress response [1,2,3]. The signaling function of H_2_S is mainly accomplished by mediating cysteine thiol persulfidation of target proteins [3,4,5,6]. Recently, protein persulfidation has emerged as a physiologically relevant modification taking place both during protein synthesis and post-translationally in a considerable fraction of the proteome in mammalian cells [6,7]. Along with thiosulfate, persulfides and polysulfides generated from proteins or low-molecular-weight thiol-containing molecules are H_2_S-derived species containing zero-valent sulfur (S^0^), commonly referred to as “sulfane sulfur”. It was recently demonstrated that these species are not only much more abundant in the cell than previously thought but also, due to their elevated nucleophilicity [8], more reactive with e.g., protein thiols or reactive oxygen species (ROS) than H_2_S or their parental thiol compounds [9]. Therefore, many of the biological effects attributed to H_2_S are currently believed to be mediated by sulfane sulfur species [10,11,12,13], whose antioxidant and cytoprotective action has been clearly established (reviewed e.g., in [14]). Moreover, sulfane sulfur species like persulfides have an increased affinity for exogenous toxins with respect to their thiol counterparts [15,16,17]. 

Steady-state levels of H_2_S and related species in blood and tissues are kept in balance through a tight regulation of H_2_S biosynthesis and catabolism [18]. In mammals, H_2_S is enzymatically produced by the two predominantly cytosolic enzymes cystathionine β-synthase (CBS) and cystathionine γ-lyase (CSE) [4] from the transsulfuration pathway and by 3-mercaptopyruvate sulfurtransferase (MST) [19]. Two isoforms of MST have been reported: MST-1, exclusively localized in the cytosol (35 kDa), and MST-2, present both in the cytosol and mitochondria (33 kDa) [20]. MST converts into pyruvate the 3-mercaptopyruvate (3MP) generated by cysteine aminotransferase, yielding a persulfidated Cys248 (human MST numbering) at the protein active site. The sulfane sulfur atom is then transferred from the persulfidated protein to an acceptor—such as cysteine (Cys), homocysteine, glutathione (GSH), dihydrolipoic acid or thioredoxin [19,21]—leading to the formation of the corresponding persulfide (R-SSH) [19], that can eventually release H_2_S. MST is therefore a recognized source of both H_2_S and sulfane sulfur species in the cell. Unlike H_2_S biosynthesis, which takes place both at the cytosolic and mitochondrial level, H_2_S catabolism occurs essentially in the mitochondrion, where sulfide:quinone oxidoreductase (SQR)—a flavoenzyme associated to the inner membrane—catalyzes the oxidation of H_2_S. The reaction leads to formation of a protein-bound persulfide [22,23] that in turn can donate its sulfane sulfur atom to sulfite or, more likely, GSH, yielding thiosulfate [24] or glutathione persulfide (GSSH), respectively [25]. SQR is thus another intracellular source of sulfane sulfur species. As SQR uses coenzyme Q as the acceptor of the electrons derived from H_2_S oxidation, SQR-mediated breakdown of H_2_S stimulates oxidative phosphorylation and, thus, ATP synthesis, which makes H_2_S the first known inorganic substrate of the mitochondrial electron transport chain [26,27]. This mitochondrial sulfide oxidizing activity varies considerably between different cell types, ranging from undetectable in neuroblastoma cells to high in colonocytes [28], the latter being physiologically exposed to the high H_2_S levels produced by the gut microbiota [29]. Indeed, Libiad and co-authors have recently demonstrated that colonic crypts at the host-microbiota interface display an apical localization of H_2_S oxidation pathway enzymes, whereas this localization pattern is lost in the epithelium of colorectal cancer [30]. 

Several human pathologies, including neurodegenerative, cardiovascular and oncological diseases, are known to be associated with altered H_2_S metabolism (reviewed in [2,6]), and the enzymes responsible for H_2_S biosynthesis are currently recognized as potential pharmacological targets [31,32,33]. H_2_S and related species, including sulfane sulfur species, seem to play a major role in cancer biology [34,35]. In colorectal cancer cell lines, CBS-derived H_2_S was suggested to support cellular proliferation, promote angiogenesis and maintain cell energy metabolism by stimulating both oxidative phosphorylation and glycolysis [36]. Accordingly, silencing or pharmacological inhibition of CBS significantly reduced cellular proliferation and migration, and tumor xenograft growth [36]. Furthermore, increased expression of CBS and CSE was documented in different cancer types, such as colorectal, ovarian, breast, prostate and melanoma, and was shown to contribute to cancer progression, energy metabolism and drug resistance [35,37,38]. MST too was found to be upregulated in different cancer types, namely, colorectal cancer [39], renal carcinoma [40], astrocytoma and melanoma [41]. However, the studies on the role of MST in cancer are scarcer and only in recent years MST, with its ability to produce sulfane sulfur species and its partial mitochondrial localization [2], was proposed as a potential target in cancer biology [42]. Recently, Libiad and colleagues reported increased SQR expression in colon cancer patient samples relatively to the surrounding normal tissue, and found a correlation between increased SQR expression and the degree of malignancy in human colorectal cancer tissues and cell lines [30]. Moreover, in the SW480 colorectal cancer cell line, an increase in mitochondrial levels of SQR has been proposed to afford an adaptive response upon exposure to hypoxia, a common feature within the tumor microenvironment [43]. 

The association between altered mitochondrial metabolism, dysregulated cell redox homeostasis and carcinogenesis is well documented (reviewed in [44]). The mitochondrial respiratory chain is a major source of ROS which are known to have opposite effects on cancer development, depending on their abundance in the cell. While at lower levels ROS can promote cancer survival and progression by causing DNA damage and activating pro-oncogenic signaling pathways [45], at excessively high levels they can induce cell death [46]. Common anticancer drugs owe their efficacy, but also their unwanted side effects, to their ability to generate oxidative stress in cancerous as well as in healthy tissues [47,48,49]. Therefore, targeting the ROS signaling pathways and redox mechanisms involved in cancer development was suggested as a potential strategy to prevent both cancer formation and the adverse effects of antitumoral drugs [50]. However, it is still controversial whether supplementation of antioxidants has an impact on limiting cancer incidence or the side effects of chemotherapy. Clinical trials have shown that antioxidants may not be beneficial and might even produce adverse effects in terms of cancer prevention and treatment (reviewed in [51,52,53]). One of the most widely used antioxidant drug is *N*-acetylcysteine (NAC), which has been extensively studied not only for its ROS scavenging action [54], but also (unfortunately, with rather disappointing results) for its presumed cytoprotective effects against anticancer drugs [55,56] and cancer preventive action [57,58,59]. Despite the wide use of NAC, the molecular mechanisms underlying the antioxidant action of this drug are not yet fully clear. Although it is generally accepted that NAC exerts its effects acting as a precursor of Cys and, thus, in turn, of GSH, it seems that the rate of intracellular deacetylation of NAC to Cys is insufficient to sustain increased GSH biosynthesis [60]. Accordingly, some studies have shown that NAC supplementation, while providing protection against oxidative stress, does not affect GSH levels [61]. Recently, Ezerina et al. proposed that NAC-derived Cys enhances mitochondrial levels of sulfane sulfur species and suggested that these species are the actual mediators of the antioxidants effects of NAC [62]. This study prompted us to investigate the effect of NAC on H_2_S metabolism in colorectal cancer cells. By combining work on the SW480 cell line and on recombinant human MST, we observed that NAC not only promotes increased expression and activity of MST and SQR, both involved in the production of sulfane sulfur species, but is also an effective substrate for MST. 

## 2. Materials and Methods

### 2.1. Materials

The human colon cancer cell line SW480 was purchased from the American Type Culture Collection (ATCC No. CCL228™). Acetyl coenzyme A (A2056), 7-azido-4-methylcoumarin (7AzC, L511455), reduced β-nicotinamide adenine dinucleotide (NADH, disodium salt, N8129), Dulbecco’s Modified Eagle Medium (DMEM) cell culture media (D6546), the cell lysis reagent CelLytic™MT (C2978), coenzyme Q_1_ (CoQ_1_, C7956), d-lactic dehydrogenase (LDH, L2395), 5,5′-dithiobis-(2-nitrobenzoic acid) (DTNB, D8130), l-cysteine (Cys, C1276), *N*-acetylcysteine (NAC, A7250), metaphosphoric acid (239275), 2-mercaptoethanol (805740), sodium 3-mercaptopyruvate dihydrate (3MP, 90374), *N*-ethylmaleimide (NEM, 34115-M), oxaloacetate (O7753), perchloric acid (PCA, 77232), protease inhibitor cocktail (P8340), rabbit polyclonal antibody against human SQR (HPA017079) and CBS (SAB1411562), anti-rabbit peroxidase secondary antibody (A6154), rotenone (R8875), sodium sulfide nonahydrate (Na_2_S·9H_2_O, 431648), sodium sulfite (S0505) were purchased from Sigma (Saint Louis, MO, USA). The bicinchoninic acid assay (BCA) kit and rabbit polyclonal antibody against human MST (PA528779) were purchased from Thermo Fisher Scientific (Waltham, MA, USA). Mouse monoclonal antibody against human CSE (SC-365381) was purchased from Santa Cruz Biotechnology (Santa Cruz, CA, USA). Fetal bovine serum (FBS), l-glutamine, streptomycin and penicillin were purchased from Biowest (Riverside, MO, USA). Mini-PROTEAN TGX Stain-Free Precast Gels, the Clarity Western ECL substrate and the Laemmli protein sample buffer were purchased from Bio-Rad (Hercules, CA, USA). Bovine serum albumin was purchased from AppliChem (Darmstadt, Germany).

### 2.2. Preparation of Sulfide Stock Solutions

Sulfide stock solutions were prepared by dissolving Na_2_S crystals in degassed ultra-pure (Milli-Q^®^, Merck Millipore, Burlington, MA, USA) water under a N_2_ atmosphere, as reported in [63]. Sulfide concentration was then measured spectrophotometrically with DTNB as reported in [64], using the molar extinction coefficient ε_412_ = 14,150 M^−1^·cm^−1^ as recommended by the manufacturer. The concentration of sulfide was then adjusted to 50 mM by dilution with degassed ultra-pure (Milli-Q^®^) water in a gas-tight glass syringe. For simplicity, sulfide solutions, though containing H_2_S, HS^−^ and S^2−^ in different proportions depending on the pH, here will be referred to as “H_2_S solutions”.

### 2.3. Cell Culture and Isolation of Mitochondria 

SW480 colorectal cancer cells were cultured in DMEM containing 4.5 g·L^−1^ glucose, supplemented with 2 mM l-glutamine, 10% (*v*/*v*) heat-inactivated FBS, 100 U·mL^−1^ penicillin and 100 μg·mL^−1^ streptomycin. For each studied condition, cells were seeded in five 25 cm^2^ flasks (1 × 10^6^ cells/flask) and grown at 37 °C and 5% CO_2_. After 24 h, the culture medium was replaced with fresh medium (control) or medium supplemented with 10 mM NAC, and further incubated for 24 h. After trypsinization, cells were washed with fresh culture medium, counted using the trypan blue dye exclusion test, and centrifuged at 1000× *g* for 5 min, yielding usually 2–3 × 10^7^ cells per condition. Under the tested conditions, 10 mM NAC proved not to be cytotoxic.

Mitochondrial preparations were obtained as described in [65] with modifications. The cell pellet was washed twice in cold PBS, resuspended in 1 mL of hypotonic buffer (3.5 mM Tris-HCl pH 7.8, 2.5 mM NaCl, 0.5 mM MgCl_2_, 1% *v*/*v* protease inhibitors cocktail) and kept on ice for 1 h. The sample was then transferred to a pre-chilled glass-teflon tissue grinder and homogenized with 100 strokes. Immediately afterwards, 100 µL of hypertonic buffer (350 mM Tris-HCl pH 7.8, 250 mM NaCl, 50 mM MgCl_2_) were added to the homogenate, which was carefully resuspended, followed by centrifugation at 1000× *g* for 5 min at 4 °C to pellet unbroken cells, debris and nuclei. The supernatant was kept on ice, while the pellet was resuspended in isotonic buffer (35 mM Tris-HCl pH 7.8, 25 mM NaCl, 5 mM MgCl_2_, 1% protease inhibitor) and centrifuged a second time at 1000× *g* for 5 min at 4 °C, to minimize heavy contaminants. The supernatants from both centrifugations were pooled and centrifuged at 20,000× *g* for 30 min at 4 °C. The mitochondria-enriched pellet was solubilized in 150 µL of 10 mM Tris-HCl pH 7.4, 250 mM sucrose, 1 mM EDTA, 0.5% (*w*/*v*) N-lauryl maltoside, as reported in [66].

### 2.4. Human MST Expression and Purification

Recombinant human MST was expressed and purified as described in Yadav et al. [19].

### 2.5. SQR Activity Assay in Mitochondrial Preparations

The SQR activity assay was adapted from [67]. Measurements were carried out in a Cary 60 spectrophotometer (Agilent Technologies, Santa Clara, CA, USA) equipped with stirring and a Peltier temperature control system. Assays were performed at 37 °C in a final volume of 1 mL. Briefly, 961 µL of 100 mM potassium phosphate buffer at pH 7.5 were poured into a quartz cuvette, covered with a 200 µL layer of mineral oil and degassed under a N_2_ atmosphere for a few minutes. The reaction mixture contained 30 µL of mitochondrial extract (typically, in the range of 37.5–139 µg total protein), 58 µM CoQ_1_, 1 mM sodium sulfite, 2 mM sodium cyanide (to inhibit complex IV) and 4 µM rotenone (to prevent H_2_S-derived reverse electron flow through complex I). The reaction was triggered by adding 96 µM H_2_S and the reaction rates were determined by following the reduction of CoQ_1_ at 278 nm (Δε_ox-red_ = 12 mM^−1^cm^−1^). Data were normalized to total protein (quantified with the BCA method) and were expressed as nmol of CoQ_1_ reduced per minute per mg of protein.

### 2.6. MST Activity Assay in Cell Lysates

The MST activity was assayed in cell lysates as described in [68] with modifications. In the presence of 3MP and a sulfur acceptor, MST produces H_2_S and pyruvate in a 1:1 stoichiometry. MST activity was therefore evaluated by quantifying the amount of pyruvate produced over a given time (1 h) by MST in the presence of an excess of the substrates. Pyruvate was quantified spectrophotometrically from the amount of NADH enzymatically oxidized in the presence of LDH. Briefly, cells were harvested by centrifugation and lysed using the CelLytic™MT reagent and the protease inhibitors cocktail from Sigma, according to the manufacturer’s instructions. Cell lysates (typically, in the range of 0.56–0.36 mg total protein) were dispensed into eppendorf tubes containing 100 mM Tris-HCl pH 8.0, 50 mM sodium sulfite, 10 mM 2-mercaptoethanol (acting as a sulfur atom acceptor [19]) and 10 mM 3MP, in a final volume of 1 mL. The eppendorf tubes were incubated at 37 °C in a thermal shaker for 1 h. Afterwards, the reaction was stopped by addition of 446 mM PCA. The sample was kept on ice for 10 min, followed by a 10-min centrifugation at 10,000× *g* at 4 °C and collection of the supernatant. Pyruvate in the supernatant was then quantified by carrying out an LDH assay in a final volume of 1.095 mL at 37 °C. The starting solution contained 100 mM Tris-HCl pH 8.0, 6.4 mM NEM, 224 µM NADH, and 50 µL of the supernatant. NEM was present to block the residual 3MP which is also a substrate of LDH [68]. NADH oxidation was triggered by adding 7U LDH and followed as an absorbance decrease at 340 nm (ε = 6.22 mM^−1^cm^−1^). Data were normalized to total protein content (quantified with BCA method) and expressed as nmol of pyruvate generated per minute per mg of protein. 

### 2.7. Recombinant Human MST Activity Assay 

Activity assays on isolated recombinant human MST were adapted from [19,33,69]. The assays were carried out in 96-well black plates, using the H_2_S-selective fluorescent probe 7AzC and a Thermo Scientific Appliskan plate reader. The reaction mixture, in 200 mM Tris-HCl pH 8.0, contained 10 μg of recombinant human MST per well, 50 μM 7AzC, and NAC (or Cys) at varied concentrations. The reaction was triggered by adding 3MP in a total assay volume of 250 μL. The increase in fluorescence (λ_ex_ = 340 nm; λ_em_ = 460 nm) was monitored over 90 min at 37 °C. Kinetic parameters for Cys and NAC were determined at concentrations ranging from 0 to 40 mM in the presence of 0.5 mM 3MP. Conversely, kinetic parameters for 3MP were determined at concentrations ranging from 0 to 2 mM in the presence of 20 mM NAC or Cys. Prior to use, the solutions of NAC, Cys and 3MP were adjusted to pH 8.0 and their concentration determined by spectrophotometric titrations with DTNB, using the molar extinction coefficient ε_412nm_ = 14,150 M^−1^·cm^−1^. Data were analyzed using Excel and the MST activity was calculated from the initial slope of the fluorescence increase.

### 2.8. Evaluation of Mitochondrial Content by the Citrate Synthase Assay

Cells were harvested and lysed as described in Section 2.6. Cell extracts were then assayed spectrophotometrically for citrate synthase in 100 mM Tris-HCl, 0.3 mM acetyl-CoA, 0.1 mM DTNB and 0.5 mM oxaloacetate, as described in [70].

### 2.9. Immunoblotting Assays

Cells were lysed as described in Section 2.6 and proteins quantified using the BCA method. Samples were separated on SDS-PAGE Mini-PROTEAN TGX Stain-Free Precast Gels (Bio-Rad, Hercules, CA, USA). The gel formulation includes trihalo compounds, which lead to UV fluorescence emission upon reaction with proteins [71], allowing to estimate the total protein load in a gel lane for normalization purposes, using a ChemiDoc MP imaging system (Bio-Rad, Hercules, CA, USA). After transfer onto a PVDF membrane, proteins were blocked for 1 h with PBS-T (phosphate buffered saline with 0.1% Tween 20 (*v*/*v*)) containing 3% bovine serum albumin (BSA, *w*/*v*) and then incubated overnight at 4 °C with antibodies against human SQR or MST. After three washing steps with PBS-T, the membranes were incubated for 1 h with horseradish peroxidase-conjugated secondary antibody, followed by three washing steps with PBS-T and detection by enhanced chemiluminescence (Clarity Western ECL Substrate, Biorad, Hercules, CA, USA). Specific bands were analyzed using Image Lab software (Biorad, Hercules, CA, USA), followed by normalization of the target protein band intensity to the total protein load determined as described above.

### 2.10. NAC Quantification by Reverse Phase High Performance Liquid Chromatography (RP-HPLC)

This method was adapted from [72]. Cells were harvested as described in Section 2.3 and washed twice in PBS. The pellet was resuspended in 250 µL ultra-pure (Milli-Q^®^) water, kept on ice for 30 min and then lysed by three cycles of freeze-thawing. Lysates were mixed with 250 µL of 3.34% meta phosphoric acid and, after vortexing, were incubated for 30 min on ice, followed by centrifugation at 12,000× *g* for 10 min at 4 °C. The supernatant was filtered and 120 µL of the deproteinized solution were mixed with 60 µL of 0.3 M Na_2_HPO_4_ and 90 µL of 1 mM DTNB. NAC quantification was performed at room temperature by injecting 100 µL of DTNB-derivatized samples into a Prevail^TM^ C8 column (150 × 4.6 mm–5 µm) (Grace/Alltech, Columbia, MD, USA) connected to a double pump HPLC apparatus (Azura ASM 2.1L; Knauer, Berlin, Germany) equipped with an UV-Vis detector (Azura ASM 2.1L; Knauer, Berlin, Germany). Injections were performed automatically using an HT300L autosampler (HTA). The elution was monitored by recording the signal at 331 nm using the following mobile phase: 20 mM NaH_2_PO_4_ pH 4.9 (Buffer A) and 100% methanol (Buffer B). The flow rate was 1 mL·min^−1^ and the concentration of buffer B was varied during the elution as follows: 2% B for 10 mL, then the concentration of B was raised to 40% in a steep gradient of 2 mL, kept at 40% for 1 mL to allow the elution of the unreacted excess of DTNB, then brought back to 2% in a 2 mL reverse gradient and kept at 2% for 7 mL before injection of the next sample. Calibrations were performed by injecting standards of NAC, GSH and Cys at known concentrations after derivatization with DTNB. For each condition, two experimental duplicates were tested, and each duplicate was injected twice. The derivatization buffer and a derivatized lysate of untreated cells were injected as controls. 

## 3. Results

### 3.1. Effect of NAC on Expression Levels of H_2_S Metabolism Enzymes 

**Figure 1:**
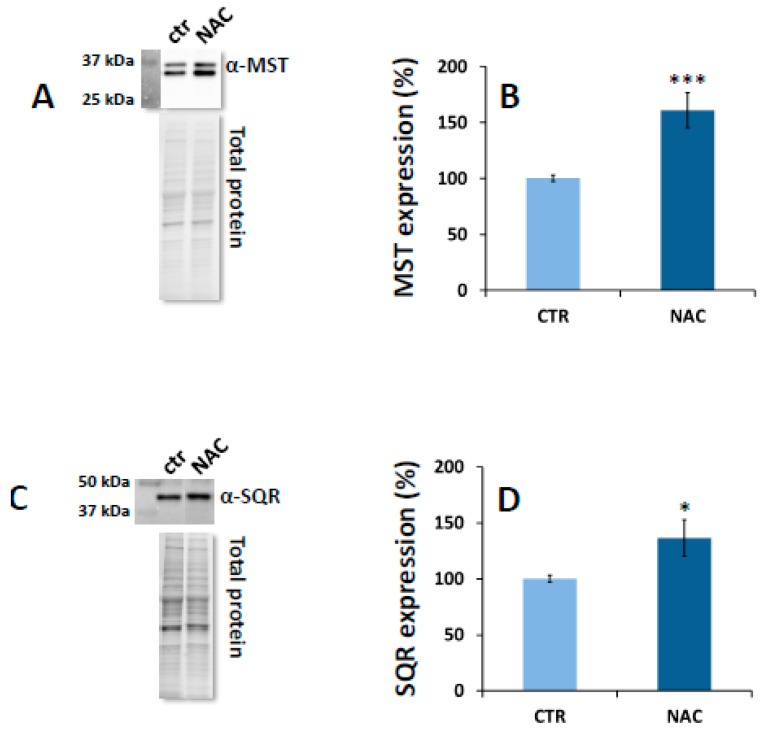
Effect of NAC on MST and SQR expression. (Panel **A**) Representative Western blot analysis of MST expression in SW480 cells grown for 24 h in DMEM alone (control) or supplemented with 10 mM NAC (NAC). The blots are shown together with their corresponding total protein load quantitation by stain-free imaging technology (see Materials and Methods). (Panel **B**) MST expression levels as normalized to total protein. Data represent the mean value ± SEM of 6 repeats, each carried out in technical duplicate. *** *p* ≤ 0.001. (Panel **C**) Similarly to panel A, representative Western blot analysis of SQR expression. (Panel **D**) SQR expression levels as normalized to total protein. Data represent the mean value ± SEM of 5 repeats, each carried out in technical duplicate. * *p* ≤ 0.05.

SW480 colorectal cancer cells were grown in DMEM alone or supplemented with 10 mM NAC. After 24 h, the expression levels of CBS, CSE, MST and SQR were evaluated in the cell lysates by immunoblotting. Whereas cell exposure to NAC had no effect on the expression of CBS and CSE (Supplementary Figure S1), as compared to control cells, NAC-treated cells displayed increased expression of both MST isoforms (overall accounting for an increment of 61 ± 16%, Figure 1A,B) and SQR (by 36 ± 1%, Figure 1C,D). In a control experiment with cells grown for 24 h in DMEM supplemented with 10 mM Cys instead of NAC, a decrease in both MST and SQR expression (by 24 ± 10% and 28 ± 9%, respectively) was observed (Supplementary Figure S2). As compared to the control, NAC did not affect cellular viability and proliferation, while treatment with cysteine proved to be cytotoxic at the tested conditions, causing impairment of cellular growth of ≈30%. 

### 3.2. Effect of NAC on MST Activity

**Figure 2:**
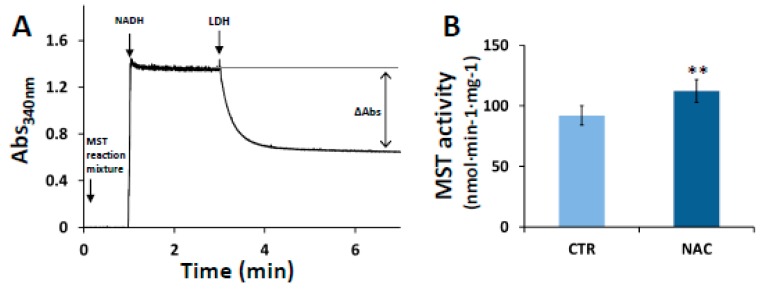
MST activity in cell lysates. (Panel **A**) Representative spectrophotometric trace of MST activity measured by LDH-coupled assay. The measurement was carried out at 37 °C under stirring conditions (see Materials and Methods). The starting solution contained the deproteinized MST activity mixture in Tris-HCl 100 mM (pH 8.0) containing 6.4 mM NEM to block unreacted 3MP. After addition of 224 µM NADH followed by addition of 7 U LDH, the pyruvate concentration was estimated from the NADH being oxidized accounting for the decrease in absorbance measured at 340 nm. (Panel **B**) MST activity normalized to total protein. Data represent the mean value ± SD of 5 repeats. ** *p* ≤ 0.01.

Prompted by the increased MST expression levels observed upon cell exposure to NAC, we evaluated the MST activity in lysates of NAC-treated cells. As described in Section 2.6, the activity was evaluated by quantifying spectrophotometrically in an LDH-coupled assay the amount of pyruvate produced over 1 h by MST in the presence of excess of 3MP and 2-mercaptoethanol. A representative trace of pyruvate quantitation is shown in Figure 2A. In the assay, the starting solution contained a diluted aliquot of the deproteinized MST reaction mixture with the produced pyruvate, and NEM to block unreacted 3MP. As expected, the absorbance at 340 nm increased after addition of NADH. Upon addition of LDH, partial oxidation of NADH was detected as an absorbance decrease, and pyruvate could be quantified from the NADH being oxidized. In line with their enhanced MST expression levels, NAC-treated cells displayed higher MST activity than control cells (112.4 ± 9.5 nmol·min^−1^·mg^−1^ vs. 92.1 ± 7.9 nmol·min^−1^·mg^−1^, Figure 2B). A blank control lacking the MST reaction mixture, and thus pyruvate, exhibited no changes in absorbance upon addition of LDH (not shown).

### 3.3. Effect of NAC on SQR Activity

**Figure 3:**
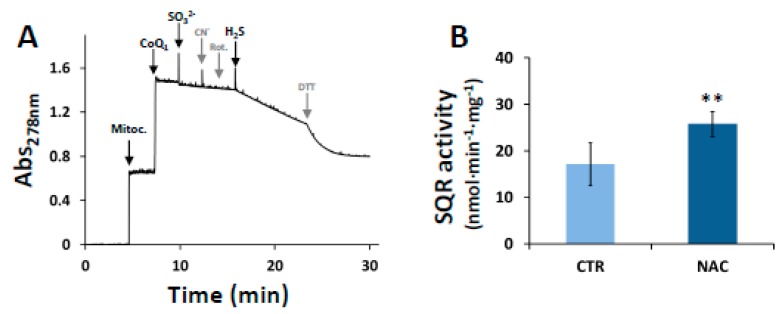
SQR activity of mitochondrial extracts. (Panel **A**) Representative spectrophotometric trace of SQR activity. The measurement was carried out at 37 °C under stirring. The starting solution contained the mitochondrial protein extract in 100 mM potassium phosphate buffer (pH 7.5) in anaerobic conditions (see Materials and Methods). Then, 58 µM CoQ1, 1 mM sodium sulfite, 2 mM sodium cyanide and 4 µM rotenone were sequentially added. SQR enzymatic activity was triggered by adding 96 µM H_2_S, and the reaction rate was estimated by following the reduction of CoQ1 at 278 nm. Afterwards, CoQ1 was fully reduced by addition of dithiothreitol (DTT). (Panel **B**) SQR activity normalized to total protein. Data represent the mean value ± SD of 5 repeats. ** *p* ≤ 0.01.

The H_2_S oxidizing activity of SQR was evaluated in mitochondrial preparations of NAC-treated and control cells by monitoring spectrophotometrically the H_2_S-induced reduction of exogenous CoQ_1_ (see Section 2.5). The efficiency of mitochondria isolation was preliminarily confirmed with immunoblotting assays revealing the presence of SQR in the mitochondrial preparations, but not (at least to detectable levels) in the cytosolic fraction (Supplementary Figure S3). A representative SQR activity assay is shown in Figure 3A. The mitochondrial preparation was preincubated with the mitochondrial electron transport chain inhibitors rotenone and cyanide, respectively to prevent re-oxidation of the reduced CoQ_1_ by Complex I through reverse electron transfer [28] or by Complex III and IV through forward electron transfer to O_2_ [67]. The SQR-mediated H_2_S:CoQ_1_ oxidoreductase activity of NAC-treated cells was 25.8 ± 2.7 nmol·min^−1^·mg^−1^, whereas that of untreated cells was 17.2 ± 4.6 nmol·min^−1^·mg^−1^ (Figure 3B). A blank control lacking the mitochondrial extract exhibited no changes in absorbance upon addition of H_2_S (not shown).

### 3.4. Effect of NAC on Mitochondrial Mass

Citrate synthase (CS) activity measurements were carried out as a validated surrogate marker of mitochondrial mass [70]. NAC-treated and control cells exhibited very similar CS activity (respectively 11.3 ± 1.4 µM·min^−1^·mg ^−1^ and 10.4 ± 1.9 µM·min^−1^·mg^−1^, Figure S4). 

### 3.5. Kinetics of H_2_S Production by Recombinant Human MST with NAC as a Substrate

**Figure 4:**
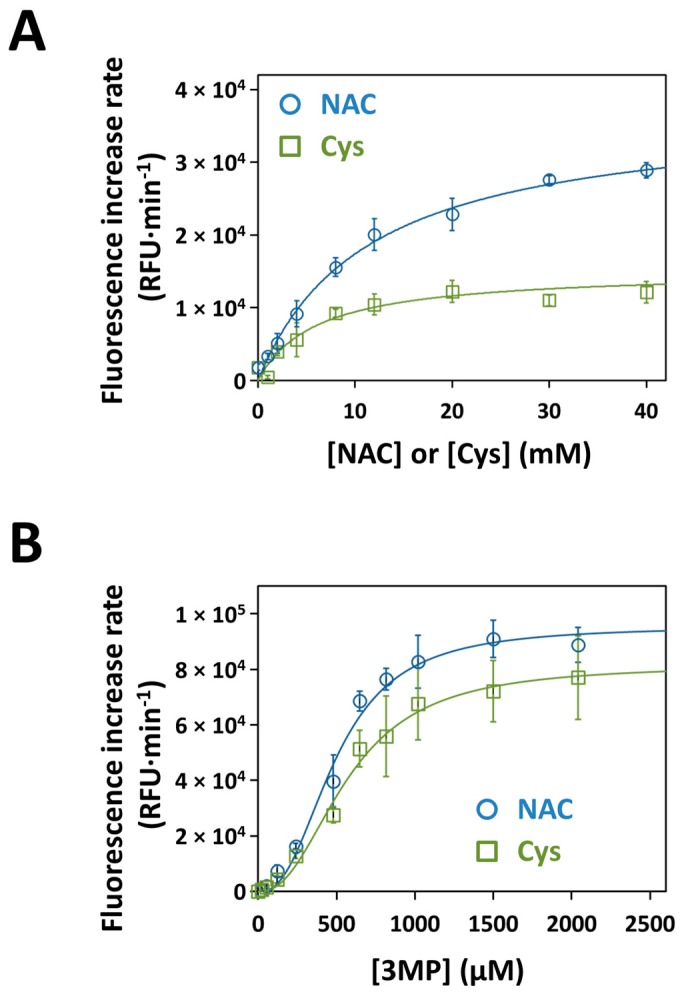
Kinetics of H_2_S generation by MST. (Panel **A**) H_2_S-synthesizing activity of MST measured at varying concentrations of Cys or NAC. The reaction mixture contained 10 μg recombinant human MST, 0–40 mM NAC (blue) or Cys (green), 50 μM 7AzC and 0.5 mM 3MP in a total assay volume of 250 μL, as described in Materials and Methods. Buffer: 200 mM Tris-HCl pH 8.0 Data represent the mean values ± SD of at least two independent experiments, each in technical triplicate. (Panel **B**) H_2_S-synthesizing activity of MST measured at varying concentrations of 3MP. The reaction mixture contained 0–2 mM 3MP, 20 mM of NAC (blue) or Cys (green). Other experimental conditions as reported in panel A. Data represent the mean values ± SD of at least two independent experiments, each in technical triplicate.

The H_2_S-synthesizing activity of recombinant human MST was evaluated in the presence of 3MP using the H_2_S selective fluorescent probe 7AzC [69]. In these assays, the ability of NAC to function as a substrate for MST was evaluated in comparison with Cys, a known physiological substrate of the enzyme [19]. The *K*_M_ and *V*_max_ values for NAC or Cys were independently determined at fixed concentration of 3MP. A hyperbolic dependence of MST activity on the substrate concentration was observed for both NAC and Cys (Figure 4A). Accordingly, data were fitted to the Michaelis-Menten model, yielding the apparent *K*_M_ and *V*_max_ values reported in Table 1. As compared to Cys, NAC yielded a 2.5-fold higher *V*_max_ and a 1.9-fold higher *K*_M_, thereby accounting for a 1.3-fold higher catalytic efficiency (*V*_max_/*K*_M_). To evaluate the effect on MST activity of the simultaneous presence of the two substrates, H_2_S production by MST was determined in the presence of Cys and NAC together. As shown in Figure S5, no significant changes in the enzyme activity were observed in the presence of both Cys and NAC, as compared to NAC alone. With either NAC or Cys at saturating concentrations, MST activity displayed clear sigmoidal dependence on 3MP concentration (Figure 4B), and both the *V*_max_ and *K*_M_ values for 3MP were in the same order of magnitude (Table 1). In these assays too, NAC was confirmed to account for a higher catalytic efficiency (1.4-fold), as compared to Cys. 

### 3.6. HPLC Quantification of Intracellular NAC 

**Figure 5:**
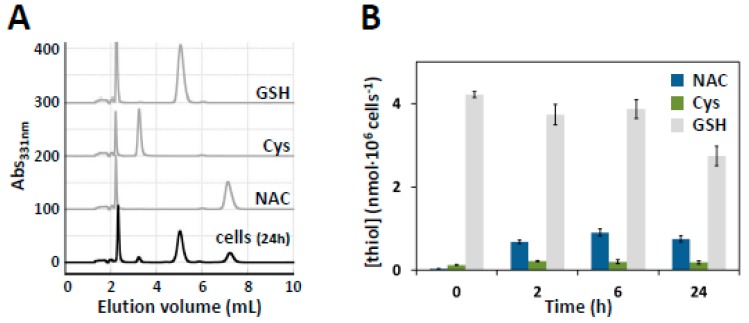
Intracellular NAC quantification by RP-HPLC. (Panel **A**) SW480 colon cancer cells were treated with 10 mM NAC for 5 min, 2 h, 6 h and 24 h. Cell lysates were deproteinized and NAC was quantified by RP-HPLC simultaneously with Cys and GSH after DTNB derivatization. Representative chromatograms are shown. Calibrations (in gray) were performed by injecting standard solutions of GSH, Cys and NAC at known concentrations. In black it is reported a representative chromatogram obtained with cells treated with NAC for 24 h. (Panel **B**) Data represent the mean values ± SD of two biological duplicates, each in technical duplicate.

SW480 cells were treated with DMEM supplemented with 10 mM NAC for 5 min, 2 h, 6 h or 24 h. After harvesting, cell lysates were deproteinized and thiol compounds were derivatized with DTNB. In each sample, NAC was determined simultaneously with Cys and GSH by using a simple and sensitive RP-HPLC method (Figure 5A), as reported in the Materials and Methods (Section 2.10). For each analyte, the calibration curves were linear at least up to 120 µM. Under the working conditions, NAC displayed good stability and a retention time (about 7 min) sufficiently distinct from those of Cys and GSH (about 3 min and 5 min, respectively). No significant interference from endogenous substances was observed at the retention times of the studied compounds. As shown in Figure 5B, under the tested conditions, the intracellular levels of NAC remained detectable and essentially constant from 2 to 24 h.

## 4. Discussion

Several lines of evidence have shown that mitochondrial dysfunction is interrelated with cancer hallmarks, such as metastatic propensity, cell death evasion, dysregulated bioenergetics and genome instability (reviewed in [44]). Altered redox balance is a common feature to cancer cells, which usually exhibit persistently high ROS levels in comparison to their normal counterparts [73]. Cancer cells are therefore endowed with an enhanced antioxidant system, which protects them from oxidative damage and likely contributes to drug resistance [74]. ROS modulation has long appeared as a possible strategy to fight cancer by either preventing malignant transformation or killing cancerous cells. However, the consequences of pharmacological ROS modulation in vivo are hard to predict [73]. While it has been proposed that targeting ROS with antioxidant compounds could prevent carcinogenesis, upregulation of ROS by anticancer drugs does suppress vulnerable cancer cells, but it also affects normal tissues, thereby contributing to the several side-effects typically associated with antitumoral chemotherapy. 

Reprogramming of sulfur metabolism has been reported as a common mechanism to different cancer types (reviewed in [2,75]). In cancer cells, enzymes involved in H_2_S synthesis, such as CBS, CSE and MST [76] were found to be overexpressed [36,37,39,40,41,77,78], and growing evidence suggests that H_2_S and related reactive sulfide species, including sulfane sulfur species, play a role in cancer biology [9,34]. While H_2_S appears to favor cancer cell survival and proliferation by stimulating cell bioenergetics and neo-angiogenesis, among other effects, sulfane sulfur species could potentiate the cell antioxidant defense system, by both scavenging free radicals and enhancing the activity of antioxidant enzymes such as glutathione peroxidase, glutathione reductase and superoxide dismutase [79,80]. (Per/poly)sulfides are indeed more nucleophilic than the corresponding thiols and their reactivity towards ROS increases with the length of the sulfane sulfur chain [14]. In this context, it is of relevance that NAC, a commonly used antioxidant drug, was recently proposed to exert its antioxidant activity precisely by promoting sulfane sulfur production [62,81,82]. Therefore, while NAC supplementation may appear as an appealing strategy to ameliorate the oxidative stress imposed by alkylating/oxidative chemotherapeutic drugs, chronic exposure to NAC may actually confer unwanted protective effects to cancer cells. 

In the present study, working on SW480 colorectal cancer cells, we explored the effect of prolonged (24 h) exposure to NAC on H_2_S metabolism, particularly on its synthases (CBS, CSE and MST) and SQR, the enzyme catalyzing the limiting step of mitochondrial sulfide oxidation. Cell exposure to NAC resulted in increased expression levels of both MST (≈60%) (Figure 1B) and SQR (≈40%) (Figure 1D), whereas neither CBS nor CSE expression levels varied significantly (Figure S1). Such changes in protein expression were not observed when cells were exposed to Cys in place of NAC under otherwise identical experimental conditions (Figure S2), which argues against the idea that the observed effects are due to the Cys derived from intracellular NAC deacetylation [83,84]. This was further corroborated by the observation that, while NAC treatment did not affect the cellular proliferation rate, exposure to cysteine resulted in ≈30% decreased cell viability. This observation neatly fits with the reported cytotoxicity of cysteine both in cell culture media [85,86] and as a dietary supplement of animal models, particularly as compared to *N*-acetylcysteine [87]. In this respect, the downregulation of both MST and SQR could be related to the cysteine-mediated cytotoxicity. 

The NAC-induced enhancement of MST and SQR expression levels points to changes in mitochondrial H_2_S metabolism. With the aim of exploring the correlation between increased expression levels and enzymatic activity, a functional approach was undertaken by measuring MST and SQR activity in cell and mitochondrial extracts, respectively. As compared to controls, NAC-treated cells displayed, respectively, ≈20% and ≈50% increase in MST and SQR activity (Figure 2B and Figure 3B), in line with the increased expression levels of these enzymes. Considering that NAC exposure did not result in changes in the mitochondrial mass (Figure S4), NAC seems to induce a mitochondrial enrichment in SQR and possibly MST, likely boosting the mitochondrial (per/poly)sulfide metabolism. In agreement with a previous report [62], we speculate that the enhanced production of mitochondrial reactive sulfide species and their physical proximity to the electron transport chain, a main source of ROS, could be at the basis of the antioxidant function of NAC. 

Besides the regulatory effect of NAC on MST and SQR expression, we tested whether NAC could stimulate the production of sulfane sulfur species by directly sustaining MST activity, acting as a sulfane sulfur accepting co-substrate, similarly to Cys or homocysteine [19]. Working on the recombinant human MST, we found that the enzyme can use NAC as a substrate to sustain H_2_S synthesis with a higher *K*_M_ but also a higher *V*_max_ when compared to Cys, overall accounting for a ≈30% higher catalytic efficiency (*V*_max_/*K*_M_) (Table 1). This higher catalytic efficiency was confirmed by measuring the enzymatic activity at varied 3MP concentrations and fixed concentration of NAC (Figure 4B; Table 1). Combined NAC and Cys acting as sulfane sulfur acceptors yielded a comparable MST activity to that measured with NAC alone, approximately the double of the activity with Cys alone (Figure S5). Therefore, NAC and Cys do not display synergic or additive effects on H_2_S generation by MST and probably compete for the same binding site on the protein. Given the higher reactivity of (per/poly)sulfides with electrophiles compared to their thiolic counterpart [15,88,89], it is likely that NAC persulfide (*N*-AceCysSSH)—the expected product of the reaction of MST with NAC—can directly contribute to ROS scavenging, as already reported for cysteine persulfide (CysSSH, [80]). It is important to note that, in addition to affording protection from oxidative stress, *N*-AceCysSSH may also increase the drug detoxifying capacity of cancer cells, thus promoting chemoresistance. Indeed, acetylation of cysteine-S-drug conjugates by microsomal NAT8 into the corresponding mercapturate is a crucial step for drug excretion in the urine [16,17]. On this basis, it is envisaged that conjugation of MST-derived *N*-AceCysSSH with an exogenous alkylating/oxidative drug will prompt the latter for its excretion. Therefore, our results suggest that NAC supplementation may help cancer cells not only evade the oxidative stress derived from anticancer drug, but even increase their drug resistance.

The physiological relevance of the reaction between NAC and MST might be questioned because cell-internalized NAC was reported to be deacetylated into cysteine [83,84], though at significant rates only in kidneys and much less in other organs [84]. To address this question, we evaluated the residual amount of NAC in treated SW480 cells by RP-HPLC at different exposure times to NAC and found that, under our working conditions, the intracellular levels of NAC remained detectable and essentially constant over at least 24 h-treatment (Figure 5B). This shows that in our experimental setting, despite possible reactions with acylases, NAC persists as such inside the cells for a considerable time, allowing its reaction with MST and other protein targets. 

**Figure 6:**
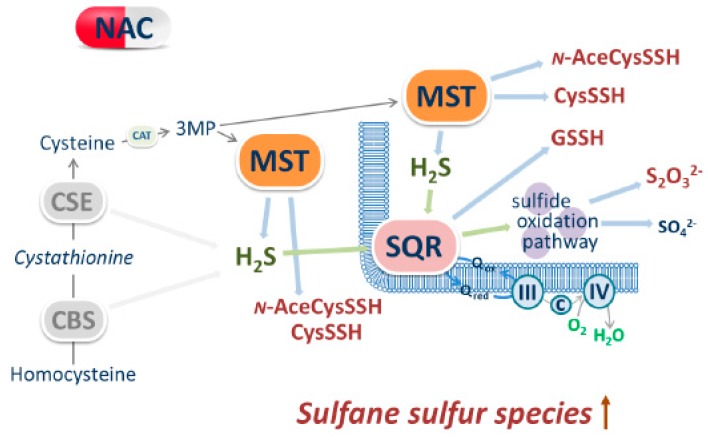
Schematic representation of the effects of NAC on H_2_S metabolism. CBS: cystathionine β-synthase; CSE: cystathionine γ-lyase; MST: 3-mercaptopyruvate sulfurtransferase; 3MP: 3-mercaptopyruvate; SQR: sulfide:quinone oxidoreductase; GSSH: glutathione persulfide; ROS: reactive oxygen species; Cys-SSH: cysteine persulfide; *N*-AceCysSSH: *N*-acetylcysteine persulfide.

In summary, this is to our knowledge the first study in which the effect of prolonged cell exposure to NAC on H_2_S metabolism has been evaluated in colon cancer cells. The evidence collected with SW480 colorectal cancer cells shows that: (i) treatment with NAC promotes increased expression and activity of mitochondrial enzymes (MST and SQR) involved in the production and consumption of H_2_S and sulfane sulfur species and (ii) NAC can act as a direct sulfane sulfur-accepting co-substrate for MST, being likely persulfidated (Figure 6). The relevance of these findings in cancer biology needs to be assessed, particularly whether the ability of colorectal cells to resist oxidative stress and develop chemoresistance could be promoted by NAC through mitochondrial metabolic reprogramming of H_2_S and related reactive species.

## Figures and Tables

**Table 1:** Kinetic parameters of human MST.

	*K*_M (A)_	*V*_max (A)_	*V*_max_/*K*_M (A)_	*K*_M (3MP)_	*V*_max (3MP)_	*V*_max_/*K*_M (3MP)_
	mM	RFU·min^−1^	RFU·min^−1^·mM^−1^	mM	RFU·min^−1^	RFU·min^−1^·mM^−1^
Cys	6.0 ± 1.0	15,136 ± 839	2523	0.57 ± 0.04	81,560 ± 3972	143,088
NAC	11.4 ± 1.0	37,178 ± 1442	3261	0.49 ± 0.02	95,396 ± 2487	194,686

(A) Values determined at varying concentration of Cys or NAC and fixed concentration of 3MP. (3MP) Values determined at varying concentration of 3MP and fixed concentration of either Cys or NAC.

## Supplementary Materials

The following are available online at https://www.mdpi.com/2073-4409/8/8/828/s1, Figure S1. Effect of NAC on CBS and CSE expression, Figure S2. Effect of Cys on MST and SQR expression, Figure S3. Effect of NAC on mitochondrial content, Figure S4. Efficiency of mitochondria isolation, Figure S5. Effect of combined NAC and Cys on MST activity.

## Abbreviations

H_2_S: hydrogen sulfide; CBS: cystathionine β-synthase; CSE: cystathionine γ-lyase; MST: 3-mercaptopyruvate sulfurtransferase; 3MP: 3-mercaptopyruvate; GSH: glutathione; Cys: cysteine; SQR: sulfide:quinone oxidoreductase; GSSH: glutathione persulfide; ROS: reactive oxygen species; Cys-SSH: cysteine persulfide; NAC: *N*-acetylcysteine; 5-FU: 5-fluorouracil; 7AzC: 7-azido-4-methylcoumarin; BCA: bicinchoninic acid; CoQ1: co-enzyme Q_1_; DMEM: Dulbecco’s Modified Eagle Medium; DTNB: 5,5′-dithiobis-(2- nitrobenzoic acid); LDH: lactate dehydrogenase; NEM: *N*-ethylmaleimide; PCA: perchloric acid; *N*-AceCysSSH: *N*-acetylcysteine persulfide.
