# Supplementary material for: N-Acetylcysteine Serves as Substrate of 3-Mercaptopyruvate Sulfurtransferase and Stimulates Sulfide Metabolism in Colon Cancer Cells
Source: Cells. 2019 Aug 4;8(8):828. doi: 10.3390/cells8080828 (PMC6721681; doi:10.3390/cells8080828)
Supplement: Supplementary file 1 [file cells-08-00828-s001.pdf]

Supplementary Material

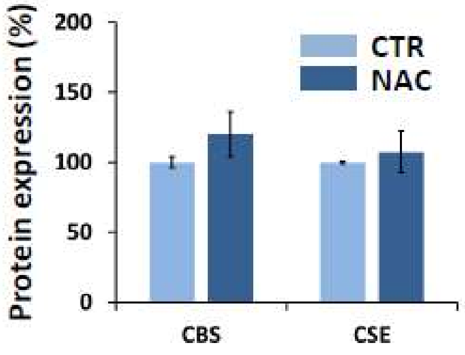

Figure S1. Effect of NAC on CBS and CSE expression. Western blot analysis of CBS and CSE expression in NAC-treated and control SW480 cells. Data represent the mean value ± SEM of 3 repeats, each carried out in technical duplicate.

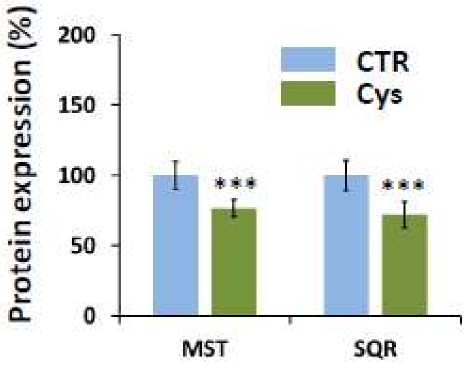

Figure S2. Effect of Cys on MST and SQR expression. Western blot analysis of MST and SQR expression in SW480 cells grown for 24 h in DMEM alone (control) or supplemented with 10 mM Cys-supplemented medium (Cys). Data represent the mean value ± SEM of 4 repeats, each carried out in technical duplicate.

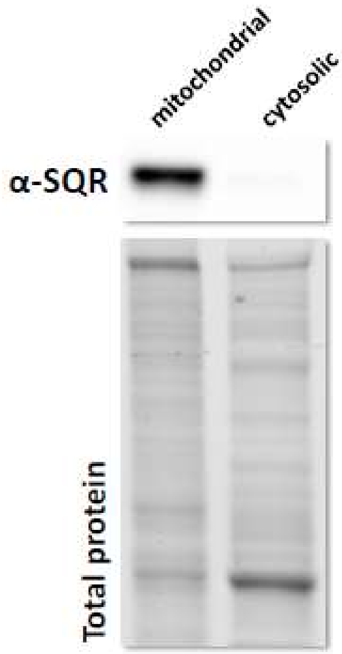

Figure S3. Effect of NAC on mitochondrial content. Mitochondrial content in NAC-treated and control SW480 cells was estimated by performing citrate synthase activity assays on cell lysates. Data represent the mean value ± SD of 5 repeats.

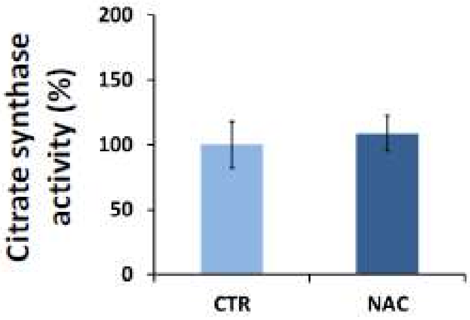

Figure S4. Efficiency of mitochondria isolation. Representative Western blot analysis of SQR to test the efficiency of mitochondria isolation. Data were normalized to their corresponding total protein load, quantitated by stain-free imaging technology (see Materials and Methods).

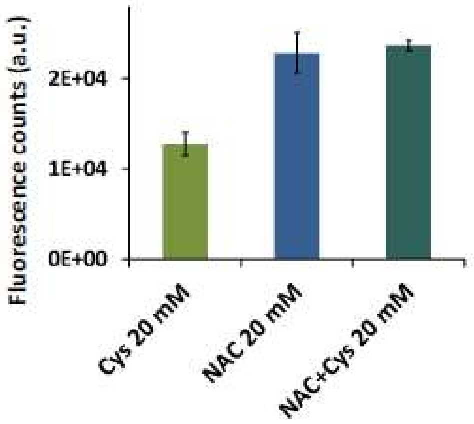

Figure S5. Effect of combined NAC and Cys on MST activity. H_2S-synthesizing activity of the recombinant human MST, as measured in the presence of 20 mM Cys (light green), 20 mM NAC (blue), or a combination of 20 mM Cys and 20 mM NAC (dark green). Buffer: 200 mM Tris-HCl pH 8.0. In addition to NAC and/or Cys, the reaction mixture contained 10 µg recombinant human MST, 50 µM 7AzC, 0.5 mM 3MP in a total assay volume of 250 µl, as described in Materials and Methods. Data represent the mean values ± SD of 3 independent experiments, each in technical triplicate.
